# Cyclic Olefin Copolymer with a Noble Metal Nanostructures as an Antibacterial Material

**DOI:** 10.3390/ijms27072940

**Published:** 2026-03-24

**Authors:** Petr Slepička, Jonáš Priškin, Bára Frýdlová, Petr Sajdl, Václav Švorčík, Anna Kutová, Petr Malinský, Zdeněk Hrdlička, Ondřej Kvítek, Nikola Slepičková Kasálková

**Affiliations:** 1 Department of Solid State Engineering, University of Chemistry and Technology Prague, 166 28 Prague, Czech Republic; 2 Department of Power Engineering, University of Chemistry and Technology Prague, 166 28 Prague, Czech Republic; 3 Institute of Physics of the Czech Academy of Sciences, Na Slovance 1999/2, 182 00 Prague, Czech Republic; 4 Neutron Physics Department of Nuclear Physics Institute CAS, Hlavní 130, 250 68 Husinec-Řež, Czech Republic; 5 Department of Polymers, University of Chemistry and Technology Prague, 166 28 Prague, Czech Republic

**Keywords:** polymer, cyclic olefin, composites, surface morphology, nanostructure, antibacterial properties

## Abstract

In this work, we demonstrate a functional and previously insufficiently explored route for converting cyclic olefin copolymer (COC) TOPAS^®^ thin films into antibacterial hybrid materials through a combination of solvent casting, plasma activation, noble-metal sputtering, and subsequent thermal or laser treatment. While COC is already well-known as a transparent, chemically resistant material for pharmaceutical and optical applications, its coupling with post-treated noble-metal nanostructures for antibacterial functionality has not been systematically described. The main contribution of this study lies in showing that COC can serve not only as a passive packaging substrate, but also as an active platform for the formation of biologically relevant surface nanostructures. Compared with previously reported metal/polymer systems, the present work provides clear evidence that noble-metal layers on COC undergo substantial structural evolution after thermal and excimer-laser treatment, resulting in regular nanoclustered morphologies. A particularly important finding is the detection of Au particle implantation below the COC surface during sputtering, as revealed by Rutherford backscattering spectrometry, which distinguishes this system from conventional surface-only metal coatings. Furthermore, we show that laser and thermal processing do not merely reshape the deposited layer, but significantly influence the final biological response of the material. Ag-based structures showed strong bactericidal behavior against both Gram-negative *Escherichia coli* and Gram-positive *Staphylococcus aureus*. The prepared samples were comprehensively characterized by AFM, DSC, RBS, SEM, and TGA, and their roughness and wettability were also evaluated, enabling direct correlation between physicochemical changes and antibacterial performance. These results introduce a new strategy for upgrading conventionally used pharmaceutical COC materials into multifunctional surfaces with added antibacterial value.

## 1. Introduction

Cyclic olefin copolymers, abbreviated as COCs, are polymers based on two monomers: 2-norbornene and ethene (also known as ethylene). This composition applies to the polymer TOPAS^®^ (an abbreviation of thermoplastic amorphous polymer [1]). COCs also offer a different selection of monomers for copolymerization: ethene and tetracyclododecene [2]. A third category within the group of cyclic olefin polymers (COPs) is prepared via metathesis polymerization of various cyclic monomers followed by hydrogenation. This method is used in the production of polymers sold under the brand name ARTON by Japan Synthetic Rubber and under the trade names Zeonex and Zeonor by ZEON Corporation [2]. Cyclic olefin copolymers (COCs) and polymers (COPs) are synthesized from the starting monomers norborene or tetracyclododecene. In the case of copolymerization with ethylene, the product is COC; in contrast, the synthesis of COP is based on homopolymerization. For the preparation of homopolymers, polymerization is carried out using a cyclic monomer, without other starting materials, via the ring-opening polymerization (ROP) mechanism, as indicated in [3]. Although COCs are advanced materials in terms of chemical resistance, transparency, etc., they are not resistant to UV radiation and cannot be used outdoors without the addition of stabilizers that absorb UV radiation and prevent degradation of the polymer matrix [3]. M. Gutiérrez-Villarreal et al. [4] studied the degradation of extruded 25 μm films made of COCs of both types (norbornene and tetracyclododecene) under 340 nm UV radiation in an accelerated climate chamber. An indisputable advantage of cyclic olefin copolymers is the greater number of applicable sterilization options [3]. Cyclic olefin copolymers are characterized by a higher glass transition temperature than pure olefin polymers. In addition to increasing the glass transition point, structural differences between ethylene units and norbornene/tetracyclododecene facilitate the preservation of the amorphous state of the solid polymer. The change in structure also results in low thermal expansion, low moisture absorption, high visible- and UV-light transmittance, and low birefringence [1].

The most common applications of COCs include microfluidics, packaging, electronics, and optics. Microfluidics is a field that deals with fluids at the micro-scale, in the range of tens of micrometers, representing the potential for miniaturization in the fields of chemistry, biology, physics, and medicine [5]. Preparation channels and microfluidic “apparatuses” from polymer materials can be acquired using the following processes [6]. Bischoff et al. [7] prepared a 200 μm deep microstructure on a COC polymer made from 1.5 mm thick COC TOPAS^®^ 6017S films using overlapping discharges of a femtosecond 353 nm laser. At the same time, they revealed the effect of beam focusing, where a defocused laser produces a rough structure on the mixer surface at powers of 4 J cm^−2^ greater than that of a focused beam. Guan et al. [8] investigated the differences in the application of PMMA- and COC-type TOPAS in the production of micropillar arrays with a diameter of 250 μm and a height of 600 μm via injection molding for microfluidic applications. They proved that COCs have better flow properties than the commonly used PMMA and thus better replicate the mold nanostructure. The advantageous chemical resistance of cyclic copolymers is used in the preparation of electro-technical components, such as single-layer transistors, from the compound MoS_2_ [9]. In the cited study, 5 nm thin films made of COC were applied to layers of silicon dioxide, which reduce the density of electron traps by a factor of 5. These are the areas that, by capturing flowing electrons, generate unreliability and errors in calculations involving the use of silicon integrated circuits and cause latency in signal detection [9]. In the packaging industry, an important property of COCs, namely, low moisture permeability, comes into play. M. Cutroneo et al. [10] investigated 10 μm COC films featuring laser-deposited 100 nm aluminum layers for packaging differentiation and a slight reduction in light transmittance to ensure a longer shelf life of food [10]. The disadvantage of using COCs in packaging is their non-biodegradable nature and non-renewable raw materials, which are in great demand today. The current alternatives are polylactic acid, polyhydroxyalkanoates, polybutylene succinate, and polycaprolactone [11].

Cyclic olefin copolymers are also applied in the field of capacitor dielectrics, including in hybrid vehicles. Z. Bao et al. [12] described the exceptional dielectric properties of COC films exposed to UV radiation in electrical capacitor applications under cyclic loading at 150 °C. The significant results obtained, with an energy charge density of 3.34 J cm^−3^ at a high voltage, were explained by the formation of carbonyl groups during the degradation of the TOPAS^®^ 6017-S copolymer matrix and the simultaneous crosslinking of the copolymer chains. The equilibrium and maximum values of crosslinking were also described when exposed to UV radiation for 10 min; with longer exposure, the material degrades significantly, and its strength decreases [12]. COCs are used as the primary packaging for drugs in the pharmaceutical industry. Drug preparations in disposable prefilled syringes made of COCs have been described, highlighting the properties of high optical transparency and surface barrier properties comparable to those of glass. COCs thus play a major role as a packaging material in the field of prefilled syringes [13]. COCs are also used in the plastic parts of blister packs. Compared to the frequently used polyvinyl chloride (PVC), COCs constitute a good alternative with better optical properties: in contrast to PVC, COCs do not lose their color over time, and they have 10 times better barrier properties against moisture [14]. Several approaches to influencing surface morphology and chemistry using either plasma activation techniques [15,16], carbon-based modification [17,18,19], laser treatment [20,21,22], or the grafting of biologically active substances have been described [23,24].

Although plasma treatment and the sputter deposition of metal nanolayers are known individually as powerful surface modification techniques, their combined use on cyclic olefin copolymers, especially with subsequent thermal or laser post-treatment, has not yet been systematically investigated. In particular, to the best of our knowledge, no detailed study has examined whether COC TOPAS^®^ films modified in this way can be transformed into antibacterial noble-metal/polymer nanocomposites, nor how the resulting structural and physicochemical changes correlate with antibacterial activity. This gap in the literature provides the rationale for the present work. The choice of COC TOPAS^®^ as the substrate was motivated by its current importance in pharmaceutical packaging, its high transparency and chemical stability, and its technological relevance as a polymer already used in medical and diagnostic applications [1,3,13,14]. If this material could be endowed with antibacterial functionality while preserving its key bulk properties, it would represent a significant advance toward multifunctional packaging and biomedical polymer systems. The choice of plasma treatment, noble-metal sputtering, and subsequent thermal or excimer-laser processing was based on the expectation that these methods would enable controllable modification of both the surface chemistry and surface morphology, while also promoting the reorganization of deposited metal nanostructures into biologically active configurations.

Therefore, the specific goal of this study was to prepare thin COC TOPAS^®^ films by solvent casting, modify their surfaces by argon plasma, deposit thin Ag and Au nanolayers, and subsequently investigate the effect of thermal and laser treatment on the resulting morphology, physicochemical properties, and antibacterial behavior. The working hypothesis was that plasma activation would increase the surface reactivity of the COC films, allowing for more effective integration of noble-metal layers, while subsequent thermal or laser treatment would induce structural reorganization of the deposited metals into nanostructured morphologies capable of enhancing antibacterial performance. At the same time, we assumed that these surface treatments would significantly influence wettability, roughness, and metal–polymer interfacial organization, which could be correlated with the final biological response. To test this hypothesis, thin solvent-cast COC films were prepared and subjected to plasma treatment, metal sputtering, thermal stress, and excimer laser exposure. The resulting materials were characterized in detail in terms of morphology, composition, thermal behavior, roughness, and wettability, and selected samples were further evaluated for antibacterial activity against both Gram-negative and Gram-positive bacteria. In this way, the present work aims not only to extend the knowledge of COC surface engineering, but also to demonstrate a new route for upgrading a conventionally used pharmaceutical polymer into a functional antibacterial material.

## 2. Results and Discussion

### 2.1. Thickness and Homogeneity of COC Films

**Figure 1:**
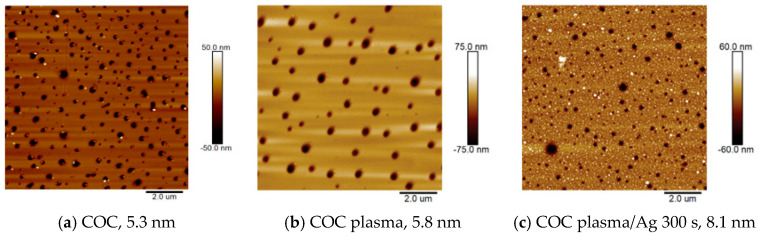
Surface morphology of the COC films (**a**) pristine, (**b**) after plasma modification for 240 s at a power of 8 W, and (**c**) after plasma modification and Ag deposition for 300 s. AFM 2D images of an area of 10 × 10 μm^2^.

Using gravimetry, the parameters of the real thickness of the films were determined using 20 randomly selected samples of pristine COC film (Figure 1a) from a series with a thickness of “100 μm”. The measurements revealed an average film thickness of 86 μm. This thickness was used in the subsequent series of experiments and exhibited the most optimal mechanical properties. The surfaces of films prepared via solvent casting are often covered with randomly placed “nanoholes”, which correspond to traces of escaping solvent nanobubbles, as shown in Figure 1b, showing the plasma-modified COC film, and Figure 1c, depicting the film after plasma modification and Ag deposition. These surface imperfections were present on the pristine films, plasma-exposed films, and films covered with metal layers.

### 2.2. Thickness of Deposited Layers

Among the parameters investigated regarding the metal layers deposited on the copolymer films was the thickness of the deposited layer. The deposition times for the gold layers were established based on a previous analysis of the sputtering rate on glass surfaces with the same current (20 mA). The sputtering times for the silver layers were determined by comparing the parameters of the calibration curves of gold and silver in order to create layers with similar heights. The thicknesses of the layers were then determined simultaneously gravimetrically and via AFM analysis, including a comparison of the thicknesses of the deposited layers of noble metals on the glass substrate. Furthermore, we denote the samples in the work in terms of the deposition time; e.g., for P/Au 293 s/T, the sample was plasma-modified, and then the Au layer was deposited for 293 s and then thermally stressed.

**Figure 2:**
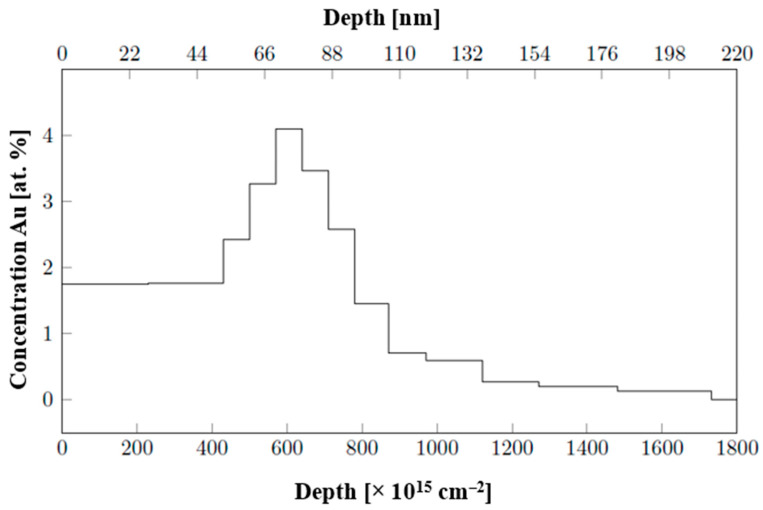
Height profile of Au atom concentration deposited for 219 s on COC foil (RBS analysis).

Relative to gravimetric analysis, determination of layer height using AFM (deposition through a mask and detection of the deposited metal–polymer interface) on COCs showed lower thickness on the samples for which Au and Ag deposition lasted longer. We did not expect this result, so we conducted an elemental analysis based on Rutherford backscattering spectroscopy. We wanted to determine whether we could incorporate noble-metal atoms into the COC foil. We confirmed that this phenomenon was achievable; thus, the thickness difference between the nanolayer and glass can be attributed to the sputtering process, where the particles penetrate the volume. This is an important result, even though it was not anticipated or planned for in our research. Similar differences were observed regarding the deposition of noble metals onto pristine and plasma-modified perfluorinated substrates [25]. However, this incorporation into the bulk directly, at energies significantly lower than usually used for ion implantation [26], surprised us, but the results of the RBS analysis in Figure 2 on COC foil onto which Au was deposited for 219 s showed the clear depth profile of the deposited layer, where Au particles were implanted under the surface of the foil, with a maximum depth of 70 nm. Therefore, the measurable COC–metal interface was evidently lower. The sputtering resulted in an enhanced sub-surface layer, where the maximum noble-metal concentration was achieved not on the very surface but at a depth of approximately 70 nm. The deposited noble-metal layers were also analyzed via EDS analysis. The results presented in Table 1 show higher noble-metal content in the samples subjected to longer deposition times, namely, 1469 s for Au particles and 2000 s for Ag particles. The EDS method confirmed the presence of residual Cl resulting from the solvent-casting method implemented.

### 2.3. Wettability

S. Roy et al. [27] described the continuous effect of the change in surface wettability after the plasma modification of COC films. According to their results, within a few days after modification, the initial effect of the reaction of the radicals that formed on and below the surface of the film with oxygen in the atmosphere takes place; at the same time, the polar groups that have formed are gradually rotated into the bulk of the materials, and thus their effect on the wettability of the material is lost [27]. Contact-angle measurements were performed for the samples before thermal stress because, as made clear in the literature, the contact angle is a time-varying parameter after plasma modification or subsequent thermal stress [15,16].

**Figure 3:**
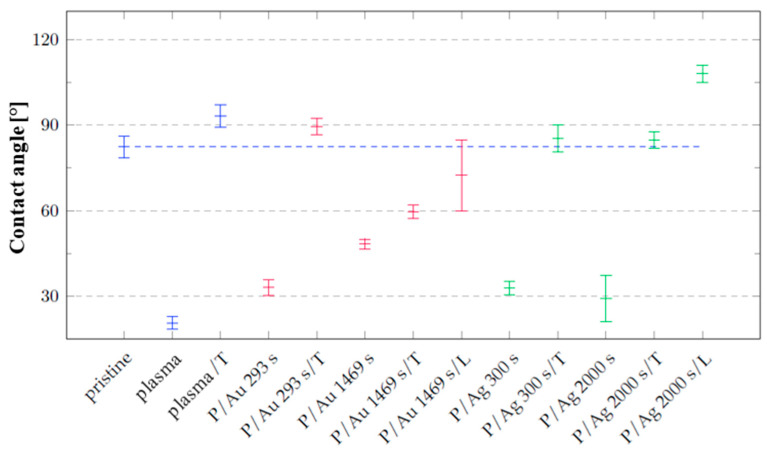
Contact-angle values of distilled water on the surfaces of the unmodified samples (pristine); the samples modified by plasma (plasma, P); the samples subjected to thermal stress (T); the samples modified using a laser KrF beam (L); and the samples onto which layers of the noble metals Au and Ag were deposited. Blue and red correspond to samples for which there was no Au deposition and samples onto which a gold layer was deposited, respectively. Green represent samples deposited with Ag.

The measurements performed immediately after modification show that after plasma modification at 8 W for 240 s, the hydrophilicity of the cyclic olefin copolymer increased, with a resulting contact angle of 20.6 ± 2.2°, relative to the primarily aged pristine COC film, with a contact angle of 82.3 ± 3.9° (Figure 3). These results are due to the reorganization of the surface bonds of the copolymer that occurred during plasma modification, mainly due to the increase in the content of radicals on the surface and the subsequent formation of, e.g., carbonyl or carboxyl groups. Oxygen atoms are incorporated into the plasma-modified surface, as described by F. Dawaymeh et al. [28]. The increase in hydrophilicity is caused by the direct reaction of radicals from the surface of the material with residual air during plasma modification and directly after modification. Another possible effect that was not studied here is the continued increase in the oxygen content on the surface for 30 days due to the presence of radicals on the surface and in the subsurface layers of the COCs, as described by S. Roy et al. [27].

A change in wettability was observed on the samples, with the minimum reached by the samples after plasma modification, where the contact angle was measured immediately after exposure to argon plasma at a power of 8 W for 240 s. In the case of the gold and silver layers, the wettability of the surface was lower than that of the plasma-modified polymer but higher than that of the pristine foil. After thermal stress was applied (as shown in Figure 3), the contact angle was comparable to that of the pristine foil for all samples, except for the sample onto which a gold layer was deposited for 1469 s. During thermal stress, the effect of polar groups on the polymer surface decreased; this effect was more pronounced only for the sample onto which a gold layer was deposited for 293 s. At the same time, the organization of metal particles on the foil surface changed. After the samples were exposed to a laser discharge at an energy of 250 mJ cm^−2^, the dispersion of the contact angle values increased for the samples covered with a Au layer, with a slight decrease in the value to 72.4 ± 12.4°, while in the case of the silver layer, the contact angle increased to 108.0 ± 3.1° after laser modification.

### 2.4. Surface Morphology

**Figure 4:**
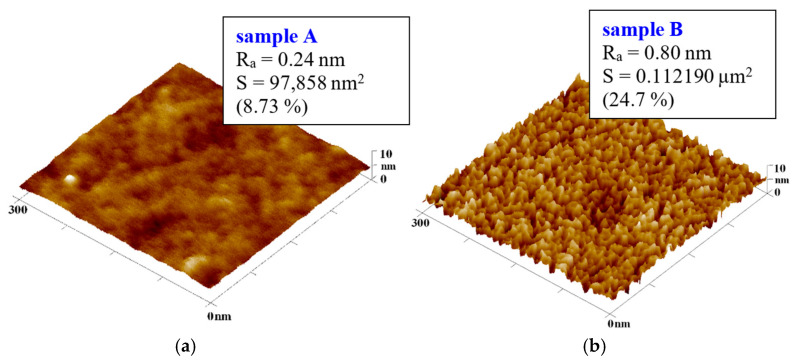
Comparison of the surfaces of a 100 μm thick COC film (**a**) before and (**b**) after plasma modification at 8 W for 240 s. 3D AFM images of an area measuring 300 × 300 nm^2^.

All stages of preparing the cyclic olefin copolymer films onto which noble-metal layers were deposited were studied using atomic force microscopy, but we first analyzed the plasma-treated COC samples. A significantly altered surface morphology induced by plasma modification was observed. The COC surface after exposure to 8 W argon plasma for 240 s showed a rough structure consisting of sharp peaks with a surface roughness higher than that of the original film, namely, Ra = 0.80 nm, while that of the pristine film was R_a_ = 0.24 nm, as shown in Figure 4.

S. Roy et al. [27] worked with COC-type TOPAS^®^ 6015 subjected to argon plasma at a power higher than 100 W. In comparison, the roughness achieved in this work was R_a_ 5.8 nm after exposure to 8 W for 240 s when measured on AFM images with an area of 10 × 10 μm^2^. These differences indicate a significant dependence of surface roughness on the performance of plasma modification. This phenomenon is due to the nature of both processes, wherein surface roughness depends on the energy required to change the arrangement of bonds on the polymer surface, while the polarity of the surface depends on the amount of oxygen available and, at the same time, the number of radicals, whose reaction with air (oxygen) subsequently creates polar groups.

#### 2.4.1. Gold Layer

**Figure 5:**
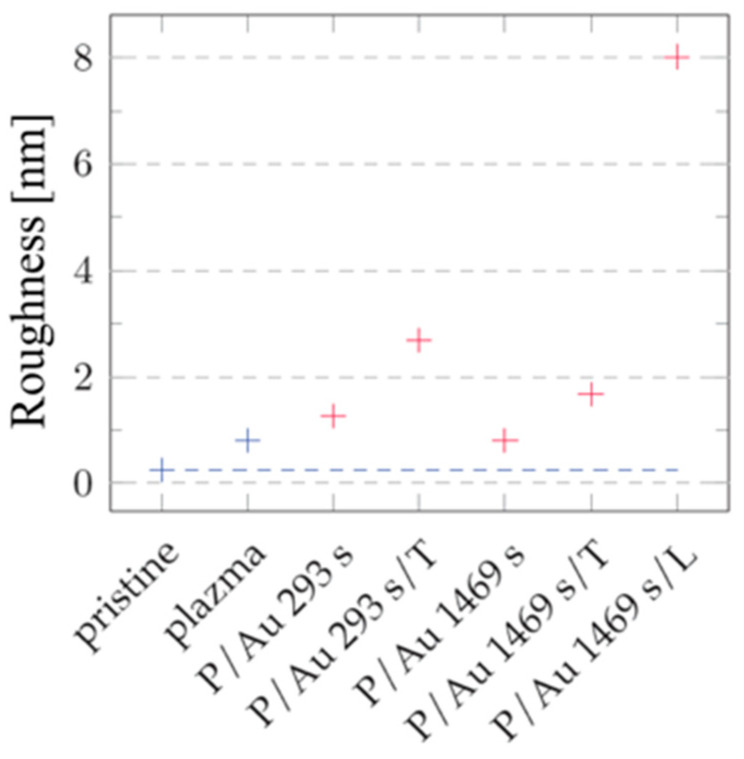
Roughness values R_a_ of the unmodified COC surface of the unmodified sample (pristine); the sample modified by plasma (plasma, P) treatment; the sample subjected to thermal stress (T); the sample treated with a laser KrF beam (L); and the sample onto which Au layers were deposited. Blue and red correspond to the samples that were not subjected to deposition and the samples onto which a gold layer was deposited, respectively. Results from AFM images with an area of 300 × 300 nm^2^ are provided.

After sputtering gold layers onto the plasma-modified surface, we observed an increase in the surface roughness value. In the case of the gold layer deposited for 293 s, the increase in roughness of the plasma-treated COC was initially moderate, rising from R_a_ = 0.80 nm to R_a_ = 1.26 nm, as illustrated in the graph in Figure 5. After exposure to thermal stress at 100 °C for 100 min, the increase was significant, rising to R_a_ = 2.69 nm. The gold layers deposited for 1469 s were subject to a smaller degree of change in roughness under thermal stress, as illustrated in Figure 5. A plasma-modified foil sample with a roughness of R_a_ = 0.80 nm after the gold layer was deposited had a comparable R_a_ = 0.81 nm. Thermal modification increased this roughness to R_a_ = 1.68 nm, while modification with a KrF 250 mJ cm^−2^ laser beam had a significant impact on roughness, which increased to R_a_ = 8.02 nm.

**Figure 6:**
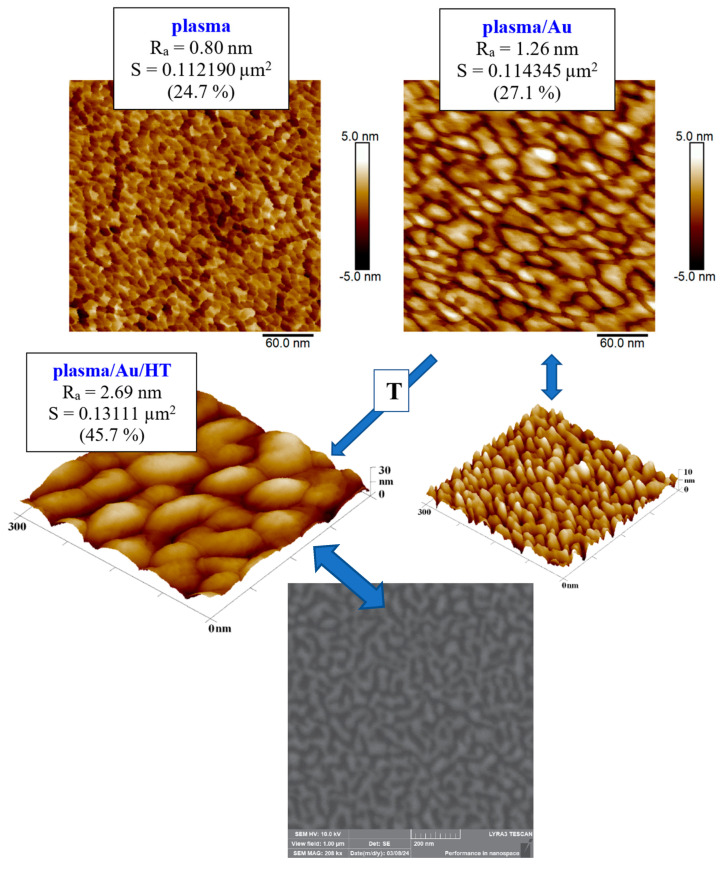
Comparison of the surfaces of plasma-treated COC foil and the same foil after undergoing 293 s of Au sputtering (plasma/Au). The COC foil with the Au layer was subsequently heat-treated (plasma/Au/HT). Evaluations are based on AFM images covering an area of 300 × 300 nm^2^. The bottom image represents an SEM scan of the plasma-treated and heated COC substrate, with a scan size of 1 × 1 μm^2^.

The difference caused by thermal stress for the film onto which a layer was deposited is visible in the AFM images in Figure 6, where nanostructures induced by plasma modification were visible on the film before it was subjected to stress, and a Au nanopattern was visible after Au deposition (the globular shapes of the structures are more visible in the 3D inset). The effect of changing the morphology of the gold surface after thermal stress on another substrate, glass plates, was studied by A. Schaub et al. [29], who described an increase in surface roughness accompanied by the formation of a globular structure for gold layers of different thicknesses, with the most pronounced increase observed for an 18 nm layer of gold particles.

This change in morphology is due to local melting of the gold nanoparticles. F. Font and T. G. Myers [30] provided a theory explaining experimental data illustrating a decrease in the melting temperature of gold nanoparticles as a function of their diameter. The data described showed a decrease in temperature to 300 K at a gold nanoparticle size of 1 nm [30]. This theory would be applicable in a case where the morphology of the gold layer on the surface of the foils is formed by aggregates of gold nanoparticles, while diffusion on the copolymer surface is realized, facilitating the conglomeration of particles.

**Figure 7:**
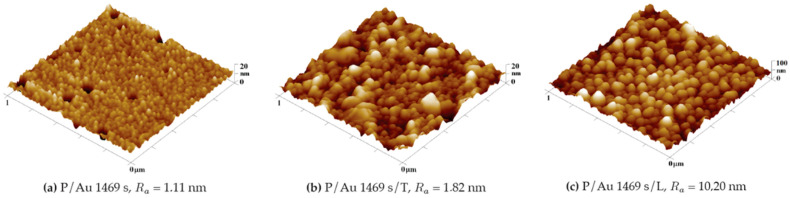
Comparison of the surface morphology (**a**) before and (**b**) after the thermal stress of a COC foil onto which a layer of gold was deposited for 1469 s and the surface of a COC foil onto which a Au layer was deposited for 1469 s after laser exposure (**c**). 3D AFM images of an area measuring 1 × 1 μm^2^.

**Figure 8:**
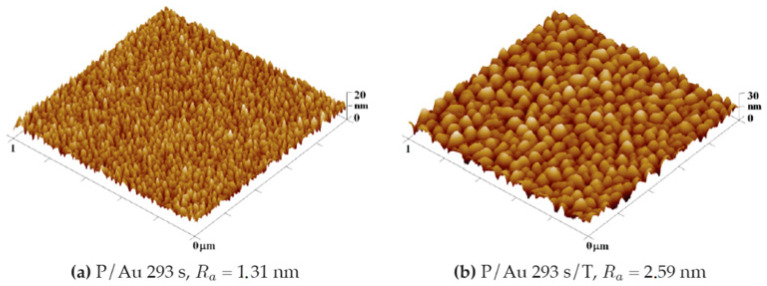
Comparison of the surface morphology (**a**) before and (**b**) after thermal stress of a COC foil onto which a layer of gold was deposited for 293 s and the surface of COC foil onto which a Au layer was deposited for 1469 s after laser exposure. These 3D AFM images correspond to an area of 1 × 1 μm^2^.

The surface morphology of the COC onto which a thicker Au layer (1469 s) was deposited also changed after undergoing thermal stress: thermal modification increased the diameter of clusters with a lower degree of fragmentation, as shown in Figure 7 and in Figure 8 for 293 s. The results clearly show the influence of the thickness of the Au layer on its roughness. On the layers deposited for 293 s, the clusters were initially separated, and with a longer deposition time, greater interconnection occurred, which reduced the fragmentation of the layer. For a description of Au layer deposition and the corresponding nucleation process, it is worth considering the study conducted by M. Schwartzkopf et al. [31] describing the influence of the deposition rate on its parameters; however, in this work, the deposition rate was the same for all layer thicknesses, and the formation of a continuous layer should therefore occur after a similar duration of deposition. A change in surface morphology also occurred when the Au nanolayer (1469 s) on the COC was exposed to a KrF laser beam with a KrF energy of 250 mJ cm^−2^, as shown in Figure 7. This exposure led to an increase in surface roughness to R_a_ = 8.02 nm.

Nanoclusters on the surface exhibited a more spherical character and lower size dispersion compared to a thermally stressed layer of the same thickness. This is due to higher energies in the form of local temperature at the moment of laser beam incidence, allowing for changes in the organization of nanoclusters. A change in surface chemistry upon exposure to a high-energy beam may also have an effect; this phenomenon affects the affinity of Au for the COC surface. In contrast, thermal modification has less potential to change the organization and chemistry of the surface. The phenomenon of the formation of more spherical, larger clusters [32] after exposure to an excimer laser is similar to what was observed in the experiments conducted by D. Q. Yang et al. [33], which directly described the increase in size and decrease in its dispersion in gold nanoparticles on the surfaces of silicon substrates after exposure to an excimer laser. In our system, the resulting morphology is undoubtedly influenced by the polymer used as well as the fact that even via simple deposition, Au atoms are incorporated into the polymer volume, as found using the RBS method (the corresponding results were introduced in Figure 2). The aforementioned study [33] also classifies the character of nanoparticles depending on the accumulated energy as the sum of individual pulses of a 248 nm laser with an energy of 20 mJ cm^−2^, demonstrating the dependence on the available energy.

#### 2.4.2. Silver Layer

**Figure 9:**
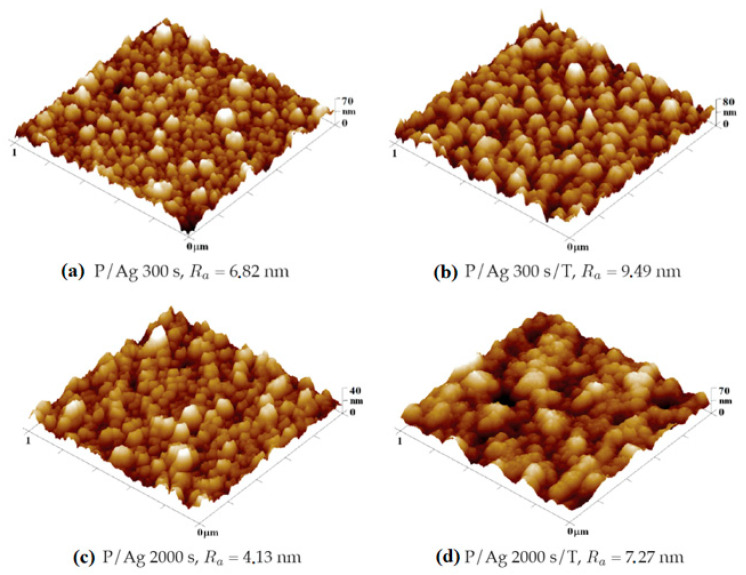
Comparison of the surface morphology (**a**) before and (**b**) after the thermal stress of COC foil onto which a silver layer was deposited for 300 s and the same set for a deposition time of 2000 s (**c**,**d**). 3D AFM images correspond to an area of 1 × 1 μm^2^.

**Figure 10:**
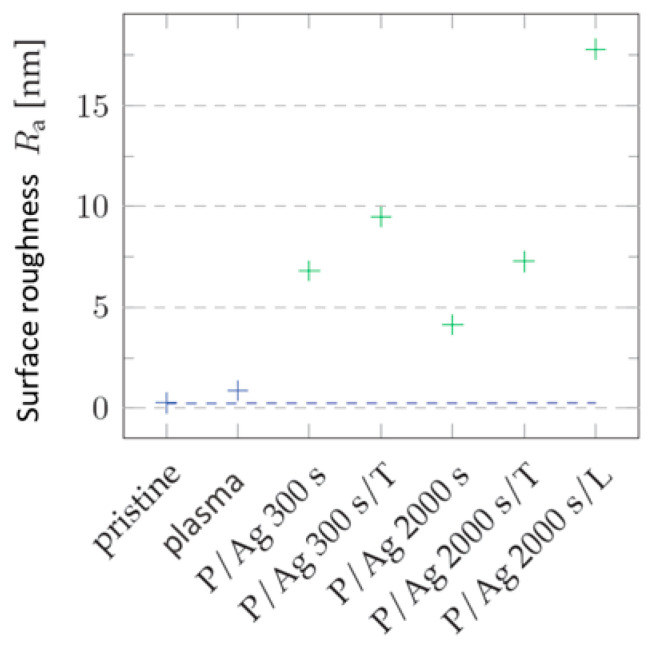
Roughness values R_a_ of COC for the surface of the unmodified samples (pristine); the samples modified by plasma (plasma) treatment; the samples subjected to thermal stress (T); the samples subjected to laser KrF beam treatment (L); and the samples onto which Ag was deposited at different thicknesses and for deposition times. Blue and green correspond to the samples that were not coated and the samples onto which a silver layer was deposited, respectively. 3D AFM images of an area of 1 × 1 μm^2^ are provided.

We analyzed the deposition of silver layers from R = 6.8 nm of the layer deposited for 300 s to R_a_ = 4.1 nm after 2000 s of deposition. During thermal stress, as with gold layers, the surface roughness increased (in Figure 9 and Figure 10) by changing the arrangement of nanoclusters into units with larger surface cross-sections, as shown in Figure 10.

In terms of shape changes, thermal stress led to the formation of globular structures in the order of tens of nm, thus highlighting the nanostructure present after metal deposition [34,35]. In the case of silver layers deposited for 2000 s, the agglomeration of Ag nanoparticles into larger clusters after thermal stress was observed again. Roughness increased from R_a_ = 4.13 nm to R_a_ = 7.27 nm. The key role in the change in surface morphology was played by the increased diffusivity of nanoparticles into the polymer surface. In the case of energy supply by exposure to a KrF laser beam with an energy of 250 mJ cm^−2^, there was a significant increase in surface roughness (R_a_ = 17.80 nm), along with a change in the shapes of the particles into segmented globular agglomerates.

### 2.5. Antibacterial Properties

Antibacterial tests against G+ and G− bacteria were performed on the prepared samples after plasma, thermal, or laser modification. The results after 2 h of cultivation did not indicate any significant effects. In comparison with preparing silver layers on the surface of polydimethylsiloxane (PDMS), for which the antibacterial activity of silver layers that were not subsequently subjected to thermal stress was observed after 1 h of cultivation, the layers on COC were probably subject to a lower release rate, indicating a higher integration of silver particles on the surface of and within the copolymer, as shown by the RBS analysis.

**Figure 11:**
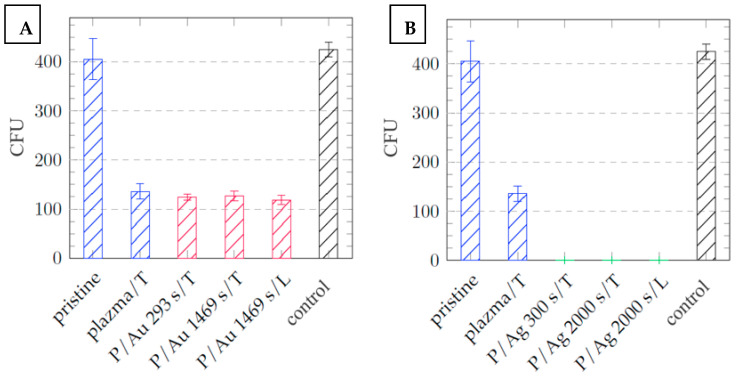
Dependence of the number of *Escherichia coli* CFUs after 24 h of cultivation on the type of COC foil sample, with layers of (**A**) gold particles = red and (**B**) silver particles = green; samples that were not covered with metal = blue; and the control = black.

**Figure 12:**
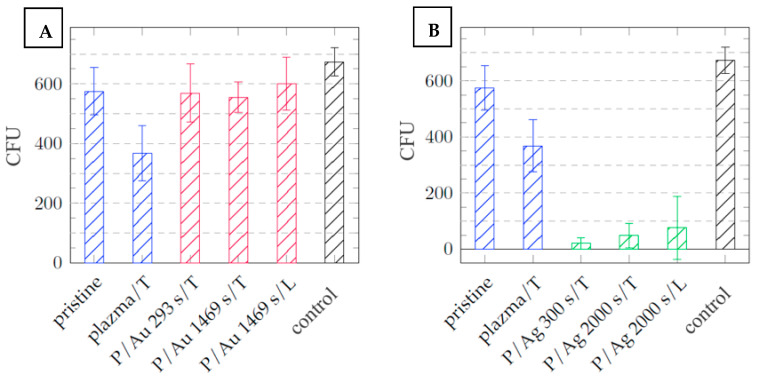
Dependence of the number of CFUs of *Staphylococcus aureus* after 24 h of cultivation on the type of sample of COC film, with layers of (**A**) gold particles = red and (**B**) silver particles = green; samples that were not covered with metal = blue; and the control = black.

After one day of cultivation, silver layers of all thicknesses exhibited significant antibacterial properties, with a decrease in the number of colony-forming units (CFUs) from 425 ± 15 and 575 ± 80 for the control and the pristine COC, respectively, to 0 for both in the case of *Escherichia coli* (see Figure 11). In the case of *Staphylococcus aureus*, significant antibacterial activity was also observed, with a decrease in CFUs for both the control and the pristine COC from 674 ± 47 and 575 ± 80, respectively, to below 100 CFUs for both (see Figure 11 and Figure 12).

Galdiero et al. [36] discussed the different modes of synthesizing silver nanoparticles (AgNPs), from their elemental state to particle form, and their mechanisms of action against multidrug-resistant and biofilm-forming bacterial pathogens. Various studies have demonstrated that AgNPs induce oxidative stress, protein dysfunction, membrane disruption, and DNA damage in bacteria, ultimately leading to bacterial death. Differences in chemical composition and stress behavior between COC and PDMS may explain the negligible difference in thermal and laser stress on the antibacterial properties of the surfaces. The increase in surface roughness after exposure to argon plasma to R_a_ = 2.20 nm contributes to the increase in the antibacterial properties of the samples that were not coated with deposited metals relative to the pristine COC film, with a surface roughness of R_a_ = 0.24 nm. In the case of *E. coli*, the decrease in CFUs after cultivation amounted to 269 ± 45 (Figure 11); for *Staphylococcus aureus*, this value was 206 ± 122 CFUs (Figure 12).

**Figure 13:**
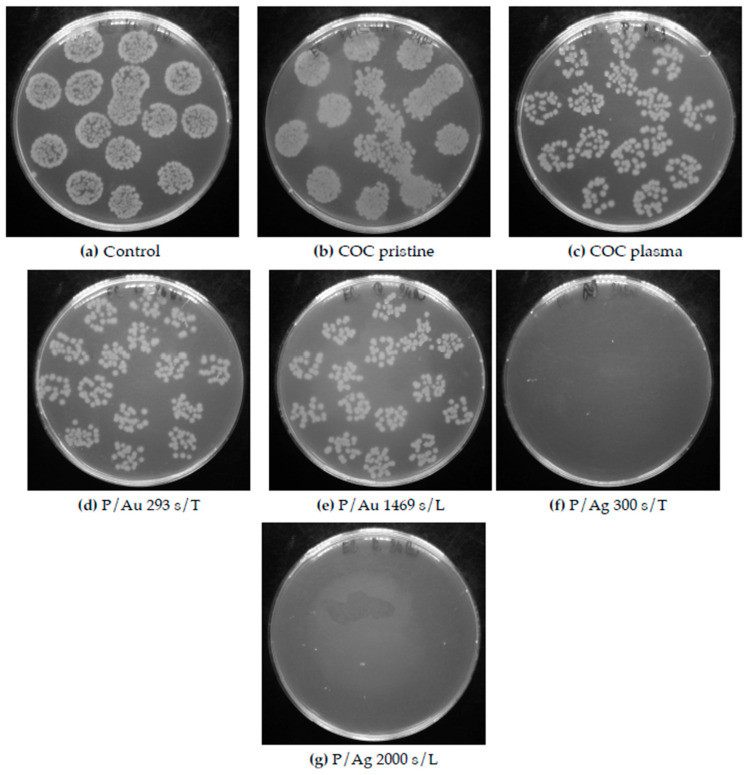
Petri dishes after cultivation of individual Escherichia coli inocula with COC foil samples. (**d**,**e**) showed low antibacterial activity of the Au surfaces, while (**f**,**g**) showed significant antibacterial activity of the Ag surfaces. COC samples: unmodified (pristine); modified by plasma (plasma, P); subjected to thermal stress (T); subjected to a KrF laser beam (L); and coated with Au and Ag layers.

**Figure 14:**
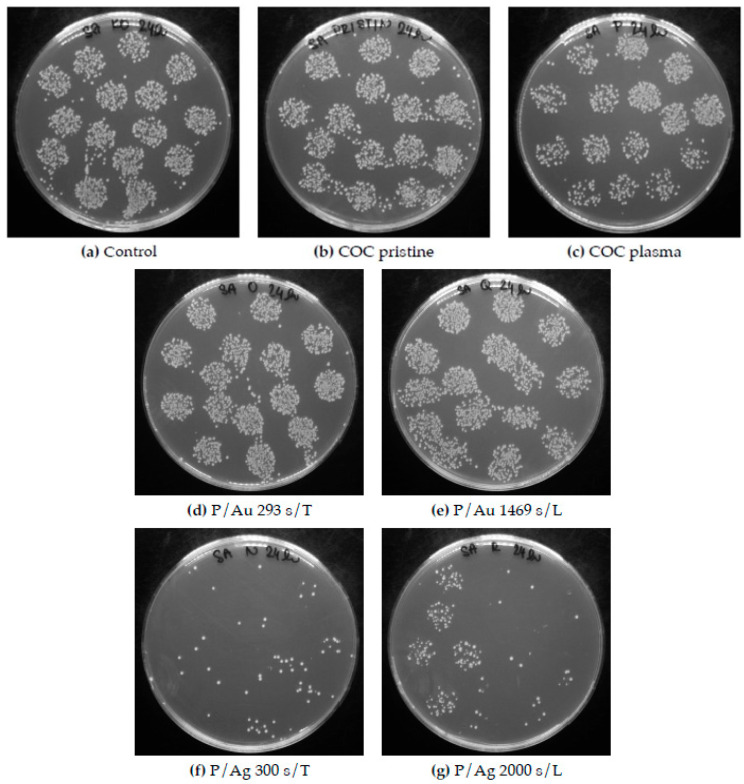
Petri dishes after cultivation of individual inocula of *Staphylococcus aureus* with samples of COC films. (**d**,**e**) show the meagre antibacterial activity of the Au surfaces; in contrast, in (**f**,**g**), a significant degree of antibacterial activity of the Ag surfaces is evident. COC samples: unmodified (pristine); modified by plasma treatment (plasma, P); exposed to thermal stress (T); subjected to a KrF laser beam (L); and coated with Au and Ag layers.

In contrast, gold surfaces had minimal effect on the activity of *E. coli* and had no effect on the activity of the Gram-positive bacterium *Staphylococcus aureus*, with potency comparable to that of the pristine foils, the plasma exposure plays a major role for antibacterial properties of these samples. It can be concluded that that the plasma treatment imparts some of the films’ antibacterial properties by altering the surface properties, such as roughness and hydrophilicity/hydrophobicity. The difference in potency between Au and Ag particles arises from the differences in the structures of the cell walls of G+ and G− bacteria: a thinner layer of peptidoglycans makes Gram-negative bacteria less resistant and results in different mechanisms of action between Au and Ag nanoparticles [37,38]. Figure 13 and Figure 14 are images of Petri dishes with cultured inoculum drops after incubation with the samples.

The antibacterial performance of both Ag and Au systems is controlled by particle size, shape, surface charge, coating chemistry, aggregation state, and the architecture of the bacterial envelope (Gram-positive versus Gram-negative) [39,40,41,42]. Ag-based systems are generally more potent because silver can undergo oxidative dissolution and continuously supply biologically active Ag+ ions, while Au is chemically more inert and therefore depends more strongly on nanoscale surface design [43,44,45]. For silver nanoparticles, the dominant antibacterial model is now considered multimodal rather than single-pathway, which is also a situation that we suspect took place for the samples that we prepared. Considering a system of silver nanoparticles, AgNPs adsorb to the bacterial surface, especially when small (roughly 1–10 nm) and positively charged or weakly stabilized, which promotes membrane contact and local structural damage. Morones et al. [39] showed size-dependent interaction of AgNPs with Gram-negative bacteria, supporting a direct surface-contact effect. Second, released Ag+ binds strongly to sulfur- and phosphorus-containing biomolecules, inactivating membrane proteins, respiratory enzymes, and DNA-associated functions. This phenomenon can also be considered to take place for Ag nanolayers, and is also responsible for the strong antibacterial properties of COC-Ag films.

Feng et al. [43] demonstrated membrane detachment, intracellular silver accumulation, and DNA condensation after silver exposure. AgNPs frequently promote reactive oxygen species (ROS) formation, causing lipid peroxidation, protein oxidation, ATP depletion, and collapse of redox homeostasis. These processes are mutually reinforcing: membrane injury enhances ion entry, while intracellular stress amplifies lethality [39,40,43,46]. Silver nanolayers/coatings act through the same chemistry, but their mechanism is shaped by immobilization on a surface. Two modes are especially important: contact killing at the interface and release killing via sustained Ag^+^ liberation. Nanolayers on implants, polymers, papers, or atomically deposited coatings reduce bacterial adhesion, damage attached cells, and may disrupt biofilm establishment. Their practical advantage is localized antibacterial action with controlled release, though performance depends on coating thickness, continuity, oxidation state, roughness, and long-term ion flux. Nanostructured silver coatings and atomic-layer antibacterial nanocoatings emphasize ROS generation, ion permeation, and contact killing as the central mechanisms [47,48]. A useful extension is bimetallic or shell-type architectures, such as silver-coated gold nanostructures, in which Au provides structural stability and Ag provides the main bactericidal chemistry [49].

Pristine AuNPs are often not strongly bactericidal because gold does not readily release toxic ions like silver; this was also confirmed in our study. However, properly designed AuNPs can kill bacteria by disrupting membrane potential, inhibiting ATPase activity, interfering with ribosomal/tRNA interactions, and serving as carriers for antibiotics or cationic ligands. A landmark study by Cui et al. [44] reported bactericidal AuNPs that collapsed membrane potential and reduced ATP levels without relying primarily on ROS. More recent work confirms that AuNP antibacterial activity is highly surface-dependent and is enhanced by cationic functionalization, anisotropic morphology, or combination with antibiotics and photo thermal activation [44,45,50]. Thus, Au is often better viewed as a tunable antibacterial platform rather than a universally strong intrinsic antibacterial material. A critical issue for both systems is selectivity. The same oxidative and membrane-active processes that injure bacteria can also damage host cells at excessive dose or prolonged exposure, especially for highly reactive Ag systems. Another concern is adaptation: bacteria can develop reduced susceptibility to AgNPs through phenotypic mechanisms such as nanoparticle aggregation mediated by flagellin, biofilm barriers, or efflux/detoxification responses. Even so, nanoparticle antibacterial action is attractive because it attacks multiple cellular targets simultaneously, making classic target-based resistance less straightforward than for many antibiotics [51,52].

## 3. Materials and Methods

### 3.1. Materials and Modification

We used the cycloolefin copolymer TOPAS^®^ 5013L-10 supplied in the form of granules by TOPAS^®^ Advanced Polymers GmbH, Raunheim, Germany. The COC produced by TOPAS^®^ is of the ethylene–norbornene type (CAS 26007-43-2). Chloroform stabilized with amylene p.a. (CAS 67-66-3) from Penta s.r.o., Prague, Czech Republic, was used for layer casting. Layer preparation using toluene p.a. was also carried out using material from Penta. The structural formula of TOPAS is [−CH2−CH2−]_m_[−CH2−CH(norbornane ring)−]_n_.

### 3.2. Solvent Casting

**Figure 15:**
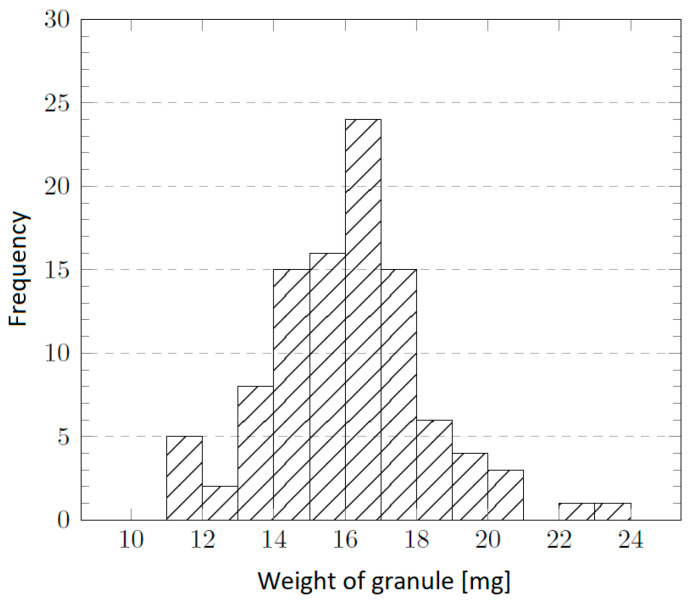
Histogram of the granule weight distribution in a sample of 100 particles.

In the sample preparation process, the parameters for “optimal” (homogeneous and uniform) foil casting were investigated first. Round Petri dishes with an inner diameter of 9 cm and an area of 63.6 cm^2^ were used as molds. From the determination of the density of the COC copolymer (1.020 g cm^−3^ [53]) and the target layer thickness, the required granulate weight for dissolution was calculated according to an equation involving COC granulate weight, Petri dish diameter, layer thickness, and COC density. The weight distribution of the TOPAS^®^ 5013L-10 granules is shown in Figure 15. Measurements were carried out gravimetrically on a random sample of 100 granules.

After the batches were weighed, a 150 mL Erlenmeyer flask and 10 mL of chloroform were prepared using a magnetic stirrer. COC granules were transferred to the chloroform through a funnel, the funnel was replaced with a stopper, and the contents were stirred for 2 h at a speed of 700 rpm at laboratory temperature. For the last 10 min of stirring, the speed was reduced to 100 rpm to stop the mixing of bubbles into the solution and, at the same time, allow the bubbles that had already been mixed in to leave the solution. This step is very important because the high viscosity of the copolymer solution after it is poured and mixed with the bubbles prevents their release.

The Petri dish into which one batch of copolymer was transferred was first cleaned with chloroform and a paper towel to prevent contamination of the bottom layer. No release agent was applied to the surface of the glass dish. The polymer films adhered well to the glass. These were poured without any irregularities forming, and after sufficient hardening, they were easily separated from the Petri dish with tweezers. After the pouring step, the solvent was evaporated on a flat laboratory table at room temperature and normal humidity. Since the COC copolymer is not known to undergo moisture absorption and the main influence on the material properties is the residual chloroform content, humidity was not monitored during the experiments. After “drying”, the layer from the glass Petri dish was transferred to a polystyrene one and cut into quarter-circular or semi-circular sections for further experiments. Initial experiments and preparation of thin metal layers were carried out on freshly prepared pieces of foil. Since the analytical evaluation of the initial DMA results showed that residual chloroform was present in the polymer even weeks after the film was cast, the samples used for the final experiments and analyses for bacterial and surface studies were prepared at least 3 months before the experiments and aged under laboratory conditions in a polystyrene Petri dish without any intervention following removal from the glass Petri dish until the first modification.

The properties of the cyclic olefin copolymer films in the initiation phase were investigated on non-standardized sectors of a circle, as this was the shape of the Petri dishes on which the films were cast. Due to the inclusion of bacterial tests, a standard sample size was adopted, and all samples were cut out with a 20 mm diameter steel cutter after the primary aging process before plasma modification. Due to the size of the culture tubes, circles with a diameter of 10 mm were punched out of the prepared samples for the bacterial study.

### 3.3. Plasma Modification

COC samples were modified on a BAL-TEC SCD 050 sputtering device in etching mode (manufacturer: BalTec Maschinenbau AG, Pfäffikon, Switzerland) using argon plasma with a power of 8 W for 240 s at a pressure of about 10 Pa. The electrode–sample distance was 50 mm. The modification was performed after the primary aging process.

### 3.4. Metal-Layer Deposition

Thin Au and Ag layers were deposited using a Quorum Q300T ES sputtering magnetron device (manufacturer: Quorum Technologies, Laughton, UK). The sputtering times were 293 s for the Au layer and 1469 s for the thicker layer. The sputtering of the silver layers lasted 300 s; for the thicker layers, the duration was 2000 s. The deposition times for all layers, including their measured thicknesses, are given in Table 2. The sputtering took place in an argon atmosphere with a pressure of 1 Pa. The deposition current was 20 mA, and the distance between the metal target and the sample was 20 cm. The samples that were deposited without undergoing the primary aging process during the initial development stage were problematic.

### 3.5. Excimer Laser Treatment

The samples were exposed to a Coherent LEAP 100K excimer KrF laser manufactured by Coherent Corp. The samples were always exposed to a single beam hit with a wavelength of 248 nm in air. Different powers were tested, but in all cases, except for the use of the highest energy, 250 mJ cm^−2^, the noble-metal layers were partially ablated. Based on the preliminary results, samples with thinner noble-metal layers than the layers deposited at 1469 s in the case of Au and 2000 s in the case of Ag were excluded from exposure to the excimer laser due to ablation. The thicknesses of the deposited metals were verified via the scratch method on glass and subsequently analyzed via AFM. The above times corresponded to 50.1 ± 0.7 nm of Au and 77.4 ± 2.0 nm of Ag on a glass substrate.

### 3.6. Heat Treatment

Before the antibacterial tests, all the samples that did not undergo laser modification were placed in a Binder oven at 100 °C for 1 h and 40 min to simulate aging after plasma modification. The samples were placed in the oven in cleaned Petri dishes. The temperature of 100 °C was chosen as the maximum temperature possible for aged samples, above which undesirable effects (bubbles, etc.) occurred during thermal stress.

### 3.7. Antibacterial Testing

Antibacterial activity was determined via a drop test using the Gram-negative bacterium *Escherichia coli* and the Gram-positive bacterium *Staphylococcus aureus*. One colony from agar plates of *E. coli* bacterial strains was transferred to 20 mL of liquid Luria–Bertani (LB) medium, and one colony from agar plates of *S. aureus* bacterial strains was transferred to 5 mL of LB medium. The inocula prepared in this way were subsequently cultured overnight at 37 °C in an orbital shaker. On the following day, the bacteria were “diluted” with sterile phosphate buffer (PBS) to concentrations of approximately 1.6 × 10^3^ bacteria per 1 mL for *E. coli* and 8 × 10^4^ bacteria per 1 mL for *S. aureus*. The tested samples were immersed in triplicate in 2 mL of bacterial suspension and statically incubated at laboratory temperature. After 2 and 24 h, the bacterial suspension was mixed, and five drops of 25 μL were pipetted onto each LB agar culture plate. These plates were cultured overnight at 28 °C for *E. coli* and at 37 °C for *S. aureus*. Subsequently, the number of colony forming units (CFUs) was determined and compared with the number of CFUs on the control and unmodified COC (pristine). This experiment was performed under sterile conditions.

### 3.8. Analytical Methods

#### 3.8.1. Atomic-Force Microscopy

The surface morphology and roughness of the pristine and treated films were examined via atomic-force microscopy (AFM) using Dimension ICON (Bruker Corp., Billerica, MA, USA). The samples were analyzed in Scan-Assyst^®^ mode using lever SCANASYST-AIR with a Si tip (spring constant of 0.4 N·m^−1^). NanoScope Analysis software 1.80 was applied for data processing. Surface roughness (*R_a_*) represents the arithmetic mean of the absolute values of the height deviations measured from the central plane.

#### 3.8.2. Scanning Electron Microscopy

The morphology of the sample surfaces was also characterized using a scanning electron microscope, namely, an FIB-SEM LYRA3 GMU (Tescan. Brno, Czech Republic). The acceleration voltage was set to 10 kV. Elemental composition was measured using energy-dispersive X-ray spectroscopy (EDS, analyzer X-ManN, 20 mm^2^ SDD detector, Oxford Instruments, Bristol, UK), while the accelerating voltage for SEM-EDS analysis was set to 10 kV.

#### 3.8.3. X-Ray Photoelectron Spectrometry

The elemental composition on the material surface was analyzed via X-ray photoelectron spectroscopy (XPS) using an ESCAProbeP spectrometer (Omicron Nanotechnology Ltd., Taunusstein, Germany). As a source, a monochromatic X-ray at an energy of 1486.7 eV was used. Atomic concentrations of the elements were determined from the individual peak areas using CasaXPS software 2.3.25.

#### 3.8.4. Wettability

The wettability of the studied samples was determined by measuring the contact angles (CA, θ) using a goniometer produced by Advex Instruments (Brno, Czech Republic) that was operated using the SEE System 7.1 program. Analysis of CA was performed at room temperature with 8 µL drops of distilled water (dyed with methyl violet) using a Transferpette^®^ automatic pipette (Brand, Wertheim, Germany) at 6 different positions of 3 samples in parallel and perpendicular directions. Subsequently, the drops were photographed and evaluated at 3 marked points.

#### 3.8.5. Gravimetry

To determine the thickness of the deposited layers, pre-weighed samples were prepared after plasma modification, metal deposition was performed, and then secondary weighing was performed. From the differences in weights, the mass of the deposited metal was calculated, and the height of the noble-metal layer was calculated through the uniform size of the sample. Weighing was performed on a UMX2 microbalance manufactured by Mettler-Toledo (Columbus, OH, USA). Due to electrostatic effects of the copolymer samples, they were discharged in an antistatic gate produced by the same manufacturer before being weighed.

#### 3.8.6. Rutherford Backscattering Spectroscopy

RBS analyses were performed using a Tandetron 4130MC accelerator at the Institute of Nuclear Physics in Řež using 1.75 MeV 4He ions. Measurements were performed with an incident angle of 0° and a scattering angle of 170°. The typical energy resolution of the FWHM spectrometer used was 15 keV. RBS spectra were evaluated using the SIMNRA code.

#### 3.8.7. Differential Scanning Calorimetry

**Figure 16:**
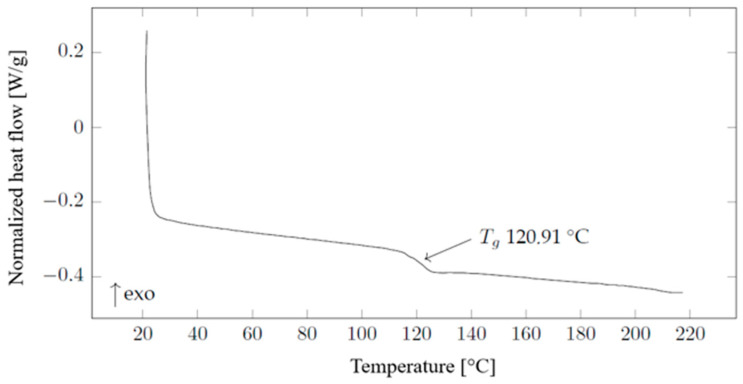
DSC measurement of the COC granulate.

Material analyses performed using the automated digital scanning calorimeter Discovery DSC250 Auto manufactured by TA Instruments (New Castle, DE, USA) were carried out in an air atmosphere from room temperature to 220 °C with a step of 10 °C min^−1^. Samples for insertion into the calorimeter binder were prepared so that their weight corresponded to 4–10 mg, and they were encapsulated in hermetic aluminum pans. An analysis of the results was carried out using the TRIOS program produced by the same manufacturer, and the method consisting of measuring half the height between the extrapolated tangents was used to determine the glass transition point. The results of the DSC analysis are presented in Figure 16. Differential scanning calorimetry measurements were performed on the granulate of the material used. The deviation in the glass transition temperature, 120.91 °C (shown in Figure 16), compared to the manufacturer’s specified temperature of 133.9 °C, may be due to the use of a different measurement or calculation procedure, since the glass transition temperature of polymers is not a discrete point but rather an interval dependent on polymer dispersity.

## 4. Conclusions

This work demonstrated that cyclic olefin copolymer (COC) can be transformed from a conventionally used passive polymer film into a functionally modified noble-metal/polymer composite with tunable surface and antibacterial properties. One of the most important outcomes of the study is the finding that Au particles are not confined only to the outer surface after sputtering, but are partially implanted below the COC surface. This result extends the current understanding of noble-metal deposition on polymer substrates and indicates that the metal–polymer interface in this system is more complex than a simple surface coating.

The present study further showed that both thermal treatment and excimer laser exposure induce pronounced reorganization of the deposited noble-metal layers, leading to substantial changes in surface morphology. In particular, laser processing of Au- and Ag-coated COC films resulted in the formation of globular nanoclusters, confirming that post-deposition treatment can be used as an effective tool for controlling the final nanostructure of the composite surface. Thus, it was fundamentally demonstrated that the morphology of noble-metal layers on COC can be actively tailored by subsequent physical processing. Plasma treatment significantly increased the hydrophilicity of the COC surface relative to the pristine foil, confirming the strong activation effect of argon plasma on this polymer. At the same time, the study showed that this induced hydrophilicity is not permanently stable and is largely lost after thermal or laser treatment in most metal–polymer combinations. The main exception was the COC foil carrying a thicker Au layer, where enhanced wettability was retained to a greater extent. This finding is important because it identifies not only the effect of plasma activation, but also the limitations of its long-term preservation after subsequent processing steps.

From the biological point of view, the most significant achievement of this work is the clear demonstration of antibacterial functionality of the prepared Ag-based nanocomposites. In comparison with the unmodified COC foil, all Ag-modified surfaces showed antibacterial activity against the Gram-negative bacterium *Escherichia coli*, with silver layers producing the strongest growth suppression. In the case of the more resistant Gram-positive bacterium *Staphylococcus aureus*, Au-based structures showed only negligible activity, whereas Ag nanocomposites again exhibited a pronounced antibacterial effect. These results confirm that silver modification is the decisive factor for achieving broad antibacterial performance in this material system, while gold plays a more limited role under the tested conditions. Overall, the study established that the combination of plasma activation, noble-metal sputtering, and subsequent thermal or laser treatment provides an effective route for engineering the surface properties of COC films. The fundamental effect achieved is the preparation of structurally controllable COC–noble-metal nanocomposites with markedly improved antibacterial behavior, particularly in the case of Ag-containing layers. These findings open the way toward the use of COC not only as a pharmaceutical packaging material with excellent bulk properties, but also as a multifunctional surface with added biological value.

## Figures and Tables

**Table 1:** Atomic concentration of elements in the prepared COC films according to EDS analysis.

Sample	C [at. %]	O [at. %]	Cl [at. %]	Au/Ag [at. %]
plasma	97.01	1.07	1.93	-
plasma/T	97.85	1.27	0.88	-
P/Au 293 s	95.61	1.11	2.52	0.76
P/Au 293 s/T	97.38	1.25	0.68	0.69
P/Au 1469 s	85.41	0.90	3.35	10.34
P/Au 1469 s/T	71.94	1.34	5.14	21.57
P/Au 1469 s/L	97.44	1.64	-	0.92
P/Ag 300 s	96.34	1.23	1.59	0.85
P/Ag 2000 s	91.01	-	2.22	6.77
P/Ag 2000 s/T	88.68	-	2.30	9.02
P/Ag 2000 s/L	97.75	0.69	1.57	-

**Table 2:** Values of the time taken to sputter the Au and Ag layers, their gravimetrically determined thicknesses (with standard deviations), and comparison with the edge height from AFM on COC and on glass (density of gold and silver: 19.32 g cm^−3^ and 10.49 g cm^−3^, respectively).

Metal	Deposition [s]	Gravimetry [nm]	AFM COC [nm]	AFM Glass [nm]
Au	293	7.34 ± 0.98	13.93 ± 0.39	13.13 ± 0.58
Au	1469	46.44 ± 2.90	10.66 ± 0.74	50.13 ± 0.67
Ag	300	6.17 ± 1.81	-	13.56 ± 0.54
Ag	2000	45.77 ± 4.79	17.07 ± 0.42	77.44 ± 2.03

## Data Availability

The data presented in this study are available at https://doi.org/10.5281/zenodo.19003188.
